# A qualitative analysis of parents’ experiences while their neonates with congenital heart disease require intensive care

**DOI:** 10.3389/fped.2024.1425320

**Published:** 2024-09-05

**Authors:** Francesca Catapano, Rochelle Steinwurtzel, Elvira Parravicini, Charlotte Wool

**Affiliations:** ^1^ Department of Medical and Surgical Science, University of Bologna, Bologna, Italy; ^2^ Department of Neonatology, Columbia University Irving Medical Center, New York, NY, United States; ^3^ Stabler Department of Nursing, York College of Pennsylvania, York, PA, United States

**Keywords:** neonatal intensive & critical care, content analaysis, congenital heart defect (CHD), parents’ experience, coping strategies

## Abstract

**Objective:**

To better understand the experience of parents with neonates with congenital heart diseases (CHD) admitted to a neonatal intensive care unit (NICU) in order to identify challenges faced by parents and discover support strategies helpful in positive coping.

**Study design:**

Prospective cohort study of parents of neonates with CHD. Parents completed a questionnaire with open ended questions regarding their experience and feeling during the hospitalization within one week of the child discharge from the NICU. Krippendorff's content analysis was used to examine data.

**Results:**

Sixty-four parents participated. Three themes were highlighted – Dialectical parental experiences, Suboptimal Parental Experiences and Positive Parental Experiences – describing the state of being and feelings that these parents face. Through this analysis, we were able to develop clinical considerations and identify coping strategies.

**Conclusion:**

The understanding of parental experience and challenges when dealing with their child admitted in the NICU is crucial to identify coping strategies to promote adaptation and enhance the development of positive coping mechanisms.

## 1 Introduction

Congenital heart disease (CHD) is the most frequent type of congenital anomaly accounting for nearly 1 percent of births annually in the United States (1); these may vary in degree of complexity, and depending on the type of malformation, the defect may require immediate medical and/or surgical intervention or a later treatment or sometimes, no treatment is needed, only follow-up is required. However, for most children born with CHD, lifelong monitoring will be required (1). Among other anomalies, CHD has the highest mortality rate, results in the longest hospital stay and subsequent frequent and prolonged hospital visits (2). These are the main explanations behind the unique and well-known psychological health pattern of parents of children with CHD (3). Despite the knowledge that all new parents can be subject to psychological distress (4), parents of babies admitted to the neonatal intensive care unit (NICU) are known to experience higher levels of stress and an altered parent-child relationships (5–9). Moreover, parents of infants with CHD admitted to Intensive Care Units (10, 11) report experiencing anxiety, stress, and post-traumatic stress disorder present from diagnosis and persistent throughout the entire course of hospitalization (12) regardless of the severity of the heart condition itself (10, 13). Numerous studies have shown that the psychological profile, often referred to as the “emotional rollercoaster”, of parents of children with CHD – depression, anxiety, somatization, hopelessness, guilt, fear, poor quality of life, hypervigilant – is a chronic state, which persists over time (12–16). Impaired parental mental health, if not properly treated, can negatively affect parents’ ability to care for their children and can eventually result in long-term cognitive, health, and behavioral problems in offspring (10, 14, 17, 18). Given the incidence of CHD, psychological distress affects a relatively large number of parents, and can lead to deterioration of the parent-child relationship. However, it has been shown that this increasingly present and persistent challenge in the Neonatal Intensive Care Unit (NICU) can be lessened by the introduction of early palliative care (PC) (11, 19).

Early PC interventions can decrease parental stress, reducing some of the unmet needs of parents and of the whole family while increasing the comfort of the newborn (19–22) but still there is a need to identify specific challenges and coping support mechanisms for parents of neonates with CHD.

The aim of this study were to better understand the experience of parents with neonates affected by CHD and admitted to the NICU in order to identify challenges faced by parents and discover support strategies helpful in positive coping.

## 2 Material and methods

### 2.1 Design

The consolidated criteria for reporting qualitative studies (COREQ) checklist was referenced to promote complete and transparent reporting, with an aim to improve rigor and comprehensiveness of the data analysis. While COREQ is specific to interviews and focus groups, and our data were retrieved via a written survey, only select items from the COREQ checklist were addressed as applicable (23).

This is a prospective cohort study of mothers and fathers of neonates with congenital heart disease admitted to the NICU at Columbia University Irving Medical Center (CUIMC) from June 2017 to May 2018. Parents of neonates transferred from other institutions, who did not speak English, or whose neonate was diagnosed with a life-limiting condition or died during the admission were excluded from the study. Approval was obtained by the CUIMC Institutional Review Board (Protocol #AAAR3403).

Parents – both elements of the couple could participate – underwent written informed consent and those who wished to participate received a printed questionnaire asking for demographic information and three open ended questions within one week of infant discharge from the NICU. An initial global question was presented to encourage parents to express their overarching experiences: “*What is it like for you to have a child in the NICU?”* This item was followed with two additional questions as follows: “*What have been the hardest parts of this experience for you?”* and “*What has helped you cope with having a child in the NICU?”*

### 2.2 Procedure

**Figure 1:**
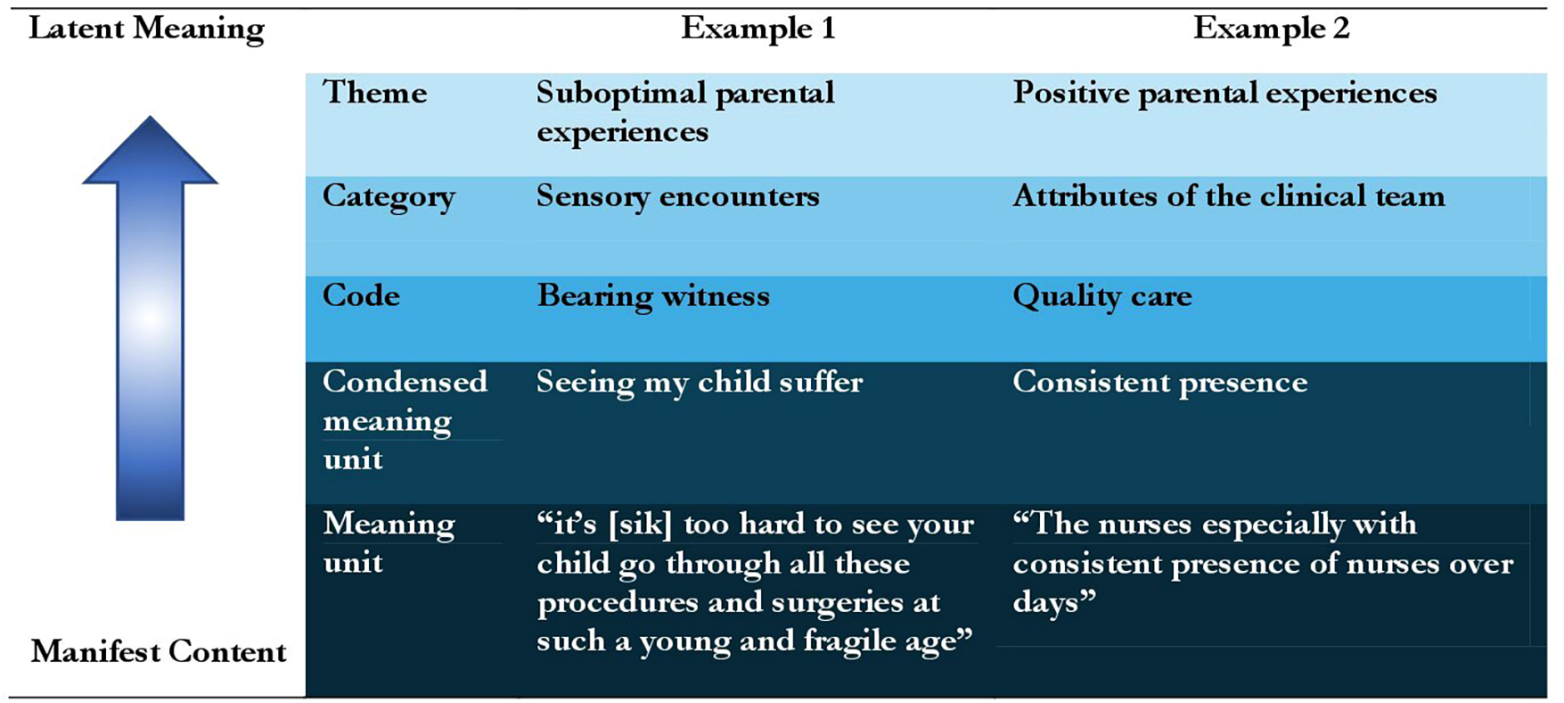
Examples of content analysis.

Krippendorff's content analysis was used to examine data, which is a research technique for the objective and systematic description of the content communicated by participants. Content analysis is context sensitive, allowing the researchers to process data texts that are significant, meaningful, informative and representational to others (24, 25). A strength of content analysis is the opportunity to increase our understanding of phenomena, in this case experiences of parents who have a neonate with a CHD in the NICU. To start, each author worked independently to examine the qualitative content for each of the three research questions. A systematic approach was used in which all authors separately mapped patterns of co-occurring words to identify clusters of common meanings. Author CW then examined the manifest data and fully quantified and coded the textual materials. Figure 1 provides examples of the analyses processes from the original data, known as manifest content, to latent meaning of the data.

These steps were followed by a meeting of all authors in which individual results were compared. A total of three themes emerged and were agreed upon. Six categories were identified by the team members, all of which were identically matched. Authors RS and CW were responsible for cross-tabulating and reexamining the data. During this meeting two additional categories were identified [Mixed and sensory] and two of the originally identified categories were merged [Gratefulness merged into positive parental feelings]. Therefore, three themes and seven distinct categories were identified. The codes within each category were carefully reexamined and when appropriate, were moved to the respective category. The entire team then verified and confirmed the final organization of the data.

A goal of the research team was to create practical clinical applications for the reader if the data support it. As such, the qualitative analysis and responses to the question “What has helped you cope with having a child in the NICU?” will be examined in order to identify specific concerns and needs of parents.

## 3 Results

### 3.1 Sample characterization

The parents of 44 neonates fulfilled the inclusion criteria. From that, 77 parents consented to participate, and, 64 (83%) submitted a written response. Demographic data are reported in Table 1 and the results are summarized in Table 2.

**Table 1:** Demographic data.

Parent demographics		*n* (%)
Gender	Mother	42 (67%)
	Father	22 (34%)
Age	<20	1 (1%)
	20–30	25 (39%)
	30–40	33 (52%)
	>40	5 (8%)
Education level^a^	Elementary	3 (5%)
	High School	8 (12%)
	College	28 (44%)
	Masters	17 (27%)
	Doctorate	7 (11%)
Marital status	Single	6 (9%)
	Married/Partnered	58 (91%)
First child	First child	25 (39%)
	Not first child	39 (61%)
Spirituality^a^	Not spiritual	17 (27%)
	Spiritual	44 (69%)
Religion^a^	Catholic	20 (31%)
	Protestant	10 (16%)
	Jewish	19 (30%)
	Muslim	7 (11%)
	Other	5 (8%)
Race^a^	Asian	3 (5%)
	African American	6 (9%)
	White	39 (61%)
	Other	6 (9%)
Neonates characteristics		
** **	Trasposition great vessels	10 (23%)
** **	Ebstein	1 (2%)
** **	Tetralogy of Fallot	5 (11%)
** **	Total anomalous pulmonary venous return	2 (5%)
** **	Coarctation	13 (30%)
** **	Arrhythmia	2 (4%)
** **	Single ventricle anatomy	11 (25%)
** **	Length of stay in days	
	Median	18
	25th/75th percentile	11/40

*N = *64 for parents and *N* = 44 for neonates.

^a^ Missing answers.

**Table 2:** Content analysis result.

Theme	Category	Code name & sample responses support the code
Theme 1 “Mixed emotions” *(n: 27)*	a. Synthesizing mixed experiences *(n: 27)*	i. Psychosocial stressors mixed with positive factors *(n: 17)* “It was quite an emotional roller coaster. Hands down, the scariest situation I’ve ever been in. It was definitely a battle between staying strong and positive vs. being “realistic” and thinking the worst. At the same time, it was incredible watching my baby improve/recover. A humbling experience”
		ii. Psychosocial stressors mixed with other variables *(n: 10)* “Unfortunate, yet needed to be done”
Theme 2 “Suboptimal parental experiences” *(n: 104)*	b. Parental stressors *(n: 72)*	i. Adverse parental feelings *(n: 42)* “Horrible. Stressful. Traumatic.” “Fear of the unknown. Length of stay and prognosis for long term.”
		ii. Separation *(n: 24)* “[Hardest part was] going home without my baby” “Heartbreaking not being with my baby 24/7”
		iii. Feeling unable to parent *(n: 6)* “Not being able to soothe my baby” “Inability to hold my child”
	c. Sensory encounters *(n: 19)*	i. Bearing witness *(n: 17)* “Watching our baby go through this” “Terrible. Caused me to have panic attacks. Dreaded sights and sounds”
		ii. Observing other babies *(n: 2)* “Seeing other babies who were sick”
	d. Concerns about balance *(n: 13)*	i. Balancing acts *(n: 13)* “Extremely difficult, helpless, sad, guilty to leave my baby, yet guilty being away from my older kids at home”
Theme 3 “Positive Parental experiences” *(n:118)*	e. Psychosocial *(n: 59)*	i. Positive parental feelings *(n: 16)* “Good because they were monitoring and checking her all the time”
		ii. Positive aspects of baby's progress *(n: 9)* “Incredible to watch [the] recovery”
		iii. Support systems *(n: 34)* “Family! So much support in every way” “Partner support” “The NICU team” “Faith”
	f. Attributes of the clinical team *(n: 56)*	i. Quality care *(n: 38)* “Knowing the nursing staff was exceptional, professional, compassionate, loving, and can take such good care of my baby” “Knowing that I was in the best hospital possible to provide exceptional care for my baby”
		ii. Team support strategies (source of Clinical Implications) *(n: 18)* “Regular updates and information” “Reached out, contacted us, asked if we needed any help” “Consistent presence of nurses over [multiple] days” “Allowed me to be here as often as I felt I wanted or needed to be” “Taught me different techniques and helpful practices” “Reassuring nurses and doctors”
	g. Coping strategies *(n: 3)*	i. Self directed *(n: 3)* “Support group” “Talking and writing about my feelings”

*n* refers to the number of times the code/category/theme was expressed in parental answers.

### 3.2 Theme 1 mixed emotions

Parents reported a variety of dialectical experiences when responding to “*What is it like for you to have a child in the NICU?”* And to “*What have been the hardest parts of this experience for you?”*. Parents face mixed emotions including happiness over small milestones, awareness and acceptance of their baby's clinical situation, stress, hope and uncertainty.

### 3.3 Category a. Synthesizing mixed experiences

#### 3.3.1 Psychosocial stressors mixed with positive factors

The psychosocial stressors parents articulated were often paired with positive factors. Several parents noted how their infant's recovery process offset their negative emotions. One parent stated:

*“It was quite an emotional rollercoaster. Hands down, the scariest situation I’ve ever been in. It was definitely a battle between staying strong and positive* vs. *being “realistic” and thinking of the worst. At the same time, it was incredible watching my baby improve/recover.”*

and

*“Mixed feelings. We are so thankful and happy that our baby made it this far. So, in some ways we are happy to be in a weird and stressful situation”*.

#### 3.3.2 Psychosocial stressors mixed with other variables

The psychosocial stressors parents articulated were often paired with other variables. Parents acknowledged their experiences as “*difficult*”, “*stressful”*, “*emotionally exhausting and traumatic”*, while recognizing their infant was “*well taken care of”* and pointing out staff attributes, including clinicians’ dedication, experience and knowledge.

*“Definitely scary but overall given the circumstances, excellent experience. Felt reassured that we were getting world class care where everyone genuinely cared for the well being of the patient.”*

A sense of practicality was also present as parents noted the need to have their infant in the NICU.

*“It was a difficult situation. On one side of the equation, I know this was the best place for him to be. A place where he could be monitored and was in the hands of experienced nurses. Yet on the other side of the equation are feelings which caused me to worry.”*

### 3.4 Theme 2 suboptimal parental experiences

Parents reported a variety of suboptimal experiences when responding to “*What have been the hardest parts of this experience for you?”* The data reported here are culled from this question as well as the first overarching question. Stressors stemmed from several feelings, including sensory encounters and the struggle to balance the demands of an infant in the NICU with other life responsibilities.

### 3.5 Category b. Parental stressors

#### 3.5.1 Adverse parental feelings

Uncertainty and fear were mentioned by many parents as some of the hardest emotional experiences. Stress and worry were also commonly expressed along with other negatively charged words. Examples of parental voice include the following: *“Horrible. Stressful. Traumatic, Very Traumatic;” “Stressful, Hurtful and painful not to go home with your baby;” “Fear of the unknown. Length of stay and prognosis for long term.”* Exhaustion was also reported by parents.

#### 3.5.2 Separation

Some parents (28%) stated the hardest part of their experience was not going home with their baby or leaving their baby behind in the NICU. The separation was described as “*heartbreaking”* and “*painful.”* Especially difficult for several parents was “*leaving at night”* and “*Being apart from my baby at night.”* Some hospital policies contributed to feelings of separation that led to dissatisfaction.

*“Not being “allowed” in the room at times. Not being able to care for my baby at times due to NICU “rules”. Having the baby be taken immediately at birth and not being able to see her four hours—seemed very unnecessary and was very upsetting.”*

Some parents felt the NICU environment contributed to a sense of separation.

“*Not being comfortable to visit, feeing like there no appropriate place to be peaceful with our baby.”*

#### 3.5.3 Feeling unable to parent

A difficult component of NICU admission is parents’ limited ability to provide the comfort and care normally given to their infants. Parents yearned to introduce the new baby to its siblings, to feed, clothe and hold the baby. Being unable to parent contributed to suboptimal parental experiences.

*“The hardest parts have been not being able to comfort my baby the way I wanted to throughout this experience…not being able to hold him nearly 2 whole weeks after birth. That was awful.”*

and

*“Feeling helpless and having to leave my baby in the NICU without my wife and I”*

and

*“The hardest part by far was the inability to hold my child. I felt as if precious bonding time was being lost.”*

Both mothers and fathers struggled with not being able to parent.

### 3.6 Category c. Sensory encounters

#### 3.6.1 Bearing witness

The sights of the NICU were distressing to many parents who described their NICU experiences as primarily visual. Parents had difficulty “*watching our baby go through this”* or “*watching him endure things such as IVs and the chest tube.”* They noted bruises, needle marks and IVs, and verbalized seeing “*my swollen baby,” “my baby on ECMO,”* and my “*baby inhibited on CPAP*.” Some parents reported dreading the sights in the NICU such as the monitors, tubes, and wires. One parent stated *“Watching him endure things such as IVs an the chest tube”* was the hardest part of the NICU experience and another parent reported *“Terrible. Caused me to have panic attacks. Dreaded sights and sounds.”* These sights and others, such as seeing their “*baby in pain”* were frequently reported as the hardest part of the NICU experience. The surgical experiences, both preoperative and postoperative, were also “*distressing to see.”* Furthermore, the sense of hearing also plays a crucial role; in fact, characteristic NICU noises such as monitor beeps have been reported to cause distress.

#### 3.6.2 Observing other babies

Seeing other children in the NICU was also reported as one of the hardest things for parents. They commented on “*seeing other babies who were sick”* and the close proximity of their infant to other babies in the NICU. Hearing the noises from other infants’ monitors was also described.

### 3.7 Category d. Concerns about balance

#### 3.7.1 Balancing acts

Parents struggled with balancing an ill child with other responsibilities. Long commutes to the NICU, parking and money were some of the items parents had to balance. Some felt unable to provide adequate support to their partner as shown in this quote: “*Balancing home, NICU, work, supporting my wife.”* Many families had children at home and struggled with splitting their time and responsibilities. One parent mentioned several challenges, all fraught with guilt: “*Extremely difficult, helpless, sad, guilty to leave my baby, yet guilty being away from my older kids at home.”* Several families simply stated they had difficulty balancing among home life, relationship with their spouse, finance, and work.

### 3.8 Theme 3: positive parental experiences

The NICU experience included positive parental experiences. When responding to the question “*What has helped you cope with having a child in the NICU?”* parents expressed many factors that positively influenced them. Support systems such as family, friends, and the clinical staff were frequently cited. Watching their infant heal and progress contributed to their coping. Parents also used self-help strategies to cope with their circumstances.

### 3.9 Category e. Psychosocial

#### 3.9.1 Positive parental feelings

Parents voiced several positive feelings when they reflected on their NICU journey. They understood the necessity of a NICU admission, and some stated they were thankful, grateful and fortunate. One parent expressed happiness stating “*We are SO thankful and happy that our babies made it this far. So, in some ways we are happy to be in a weird and stressful situation.”*

#### 3.9.2 Positive aspects of baby's progress

The opportunity to see their child progress and heal contributed to coping and positivity. One parent stated their “*sick child was getting better”* while another reported that it was “*incredible to watch recovery.*” Several parents were grateful their child was given a “*chance to survive”*.

#### 3.9.3 Support systems

Parents relied on many different supportive relationships. Support from family and the nursing staff were most heavily mentioned (33% and 31% respectively). Spouses expressed appreciation for their partners. Parents voiced appreciation for supportive friends. Seventeen percent of respondents accessed their faith in God and prayer to help them cope.

### 3.10 Category f. Attributes of the clinical team

#### 3.10.1 Quality care

Parents repeatedly expressed high regard for the clinical team members. Nurses, physicians, and other professionals were considered integral to the provision of quality care. Clinicians were described as “*amazing”, “qualified”, “experienced”, “friendly”,* and “*knowledgeable.”* Families felt a sense of relief their baby was in good hands and were “*confident in the team.”* One parent stated two quality attributes:

*“1.) Knowing the nursing staff was exceptional, professional, compassionate, loving, and can take such good care of my baby 2.) Knowing that I was in the best hospital possible to provide exceptional care for my baby.”*

Families benefitted from team members who provided comfort to them as parents and to their infant and expressed good care helped them to cope.

#### 3.10.2 Team support strategies

Parents provided data that reflect the value of specific clinician behaviors, such as “*Consistent presence of nurses over [multiple] days”* and “*Reassuring nurses and doctors”*.

### 3.11 Category g. Coping strategies

#### 3.11.1 Self directed

Parents accessed several tools available to support their adaptation. While one parent preferred “*taking one moment at a time”* others preferred “*preparing in advance at pre-diagnosis”* and researching the condition. Parents found talking to other mothers, attending hospital support groups, and writing about their feelings useful. Some held a belief their baby would heal. Just being with and holding the baby were considered useful coping strategies.

Consideration for clinical practice in order to support parental coping are reported in Table 3.

**Table 3:** Strategies to support parental coping and considerations for clinical practice based on anwsers to question “what has helped you cope with having a child in the NICU?”.

Strategies to support parental coping	Data from parental responses	Considerations for clinical practice
Knowledge and education	*“Knowing diagnosis and treatment options ahead of time prepared me for what I can expect my baby to be treated with how long processes will be.”* *“Always took the time to explain to us what was going on.”* *“Regular updates and information.”* *“Reached out, contacted us, asked if we needed any help.”* *“Taught me different techniques and helpful practices.”*	• Assess parental knowledge prior to providing information. • Assess parental desire for knowledge – do they want every detail, or do they prefer small pieces of information over time? • Provide information in a timely fashion. • Consider contacting parents to offer support as appropriate. • Role model best practices. • Educate parents on evidence-based physical and developmental care for their infant.
Family presence in NICU	*“Being at daily rounds also helped immensely to fully understand what was going on and plan for the day.”* *“Allowed me to be here as often as I felt I wanted or needed to be.”*	• Invite parents into the clinical environment as partners on the care team. • Allow 24/7 parental involvement. • Provide a sleeping area for parents close to their infant.
Reassurance	*“Reassuring nurses and doctors.”* *“Talked us down and told us everything is going to be alright.”* *“Nurses telling me it is ok.”* *“Positive smiling faces.”* *“Helped cope with my son.”*	• Assess parental feelings. • Cultivate an environment of encouragement. • Support the changing hopes and dreams of parents. • Offer emotional support and kind words. • Provide resources that support therapeutic coping mechanisms.
Focus on quality care	*“The quality of care provided.”* *“Experienced staff.”* *“Consistent presence of nurses over days.”* *“Exceptional, professional, compassionate, loving, and can take such good care of my baby.”* *“Help and support everyone gives here.”* *“Knowing how caring and supportive the nurses were to him.”*	• When able, schedule consistent caregivers. • Maintain professionalism coupled with genuine care. • Assure parents their infant is in experienced hands. • Early Palliative Care • Family Centered Care
Provision of resources	*Staff “to help with resources and dealing with the stress and strain.”*	• Provide evidence-based, current resources to parents such as books, brochures, websites, and community supports. • Psychoeducational groups

## 4 Discussion

The main finding of our research is the identification of effective clinical coping strategies drawn from the experiences of parents with children undergoing hospitalization in the NICU due to Congenital Heart Disease (CHD). We have detailed these strategies for supporting parental coping in Table 3, along with qualitative data that underscores their effectiveness and considerations for clinical application. This achievement was made possible by conducting a thorough Krippendorff content analysis.

Theme 1 “Mixed emotions” refers to the different and opposing emotions that parents of CHD children face, consistent with other studies in this population (12–16). Our cohort of patients showed that their psychological asset is characterized by stress, fear, and anxiety. Alongside this vortex of negativity come the positive elements such as hope, acceptance, the joy of seeing their child fighting, getting better and making progress. Indeed, sometimes they expressed sadness and desperateness, other times they felt hopefulness and happiness: their emotions are not linear, they experience ups and down. This rollercoaster of emotions emphasizes how all parents of infants with CHD are at high risk for psychological distress (20, 26). We believe that in caring for these children and thus consequently for their parents, we cannot forget the psychological profile of these parents (27). We emphasize as the main clinical strategy associated to this first theme the continuous assessment of parental feelings and the support in the changing in hopes and dreams of parents. Indeed, health care professionals can have a significant impact on the care of these parents, embracing an individualized approach that supports the unique needs of families at different times (17, 28, 29), reassuring them and adapting care strategies according to either “Suboptimal Parental Experiences” (Theme 2) or to “Positive Parental Experiences” (Theme 3). These two themes identify the two alternating psychological states that are characteristic of the psychological set-up described so far. Thus, Theme 1 is composed of an alternation of Theme 2 and Theme 3, which we will analyze in more detail below.

Theme 2 “Suboptimal Parenting Experiences” is characterized by negative parental feelings. Parental stress appears in our cohort to be mainly due to separation from their child and the subsequent feeling of being unable to parent, which is consistent with prior work (11, 19). Indeed, in this regard, parents emphasized as coping mechanisms the ability to be with their child as much as possible and the need to feel involved in health care decisions. We hold that it is necessary and of utmost importance the opportunity for parents to be with their babies 24/7 by providing a space where they can rest and at the same time be next to their newborn. It is critically important to build a relationship of trust with the care team so that parents feel that they are part of the health care decisions about their child's health, regardless of the infant's medical fragility. Establishing a solid relationship of trust (9) will facilitate a conversation with the healthcare team in order to explore the family's goals, hopes and concerns, allowing them to regain a sense of control. Fundamental is the implementation of early PC. PC is not only effective in treating conditions defined as life-limiting (30–32) or serious illnesses, such as CHD, but it is essential where survival is still a likely outcome (21). PC helps parents regain their sense of parenthood by bonding, holding, touching, feeding and taking care of their child (19, 33, 34). PC has also been shown to decrease anxiety in parents of newborns with CHD and it was also found that PC interventions helped parents with other children suggesting possible help in balancing all their responsibilities (20). Indeed, an additional category we identified in Theme 2 is the one that concerns loss of balance leading to a feeling of guilt, not only about the sick child, but also the siblings, the partner, the difficulty of managing these relationships, finances, and work. In correlation with this, what parents reported as most helpful was the presence of the partner, family members and friends (Theme 3 category support system); thus, our data shows that a family centered care approach is necessary (26, 33, 34).

The last category belonging to Theme 2 is sensory encounters, including the sight of their own child going through a serious and painful journey, the presence of other children suffering and the environment (sounds, monitrs etc) of the NICU. Negative sensory encounters can lead to trauma and post-traumatic stress syndrome (8, 35, 36). For this category, no coping skills were evidenced by the parents, and we consequently could not identify clinical strategies as the suffering described is part of the pathway of the conditions under consideration. Since most diagnoses of CHD are done prenatally, it maybe helpful explaining to the parents what their baby will look like during the NICU admission and at the time of surgery. Pictures can be shown along with explanation of the meaning of wires, tubes, and lines that will be placed on the baby's body. Moreover, a prenatal tour of the NICU may help visualize the reality of the care and the hardware needed to help the baby during the admission.

Theme 3 “Positive parental experiences” is characterized by positive parental feelings arising from three main categories. In the psychosocial category, we identified the positive emotional parental feelings resulting from the acceptance and understanding of the need for hospitalization, their child's progress, and gratitude to the support system. In the support systems we identified the partner, family, faith, and healthcare team. As stated above family and partner are the most cited as coping mechanisms in our sample. The medical care team deserves further consideration as the quality of care reported by the parents interviewed enables the creation and maintenance of a trusting relationship, which is fundamental as reported at the beginning of the discussion. In addition, the key role of the team that was able to support the parents in this journey and help them face the hospitalization and care of their child, as reported in the literature (28, 37), emerged as one of the best coping support mechanisms. Other coping strategies identified in the analysis include self directed (Category g.i – Theme 3), such as journaling to express feelings, as well as participation in support and psychoeducational groups – these strategies have been demonstrated to enhance the quality of life of parents with CHD newborns (27).

Strengths of this study include the extensive sample size analyzed, particularly noteworthy for a qualitative analysis. The Krippendorff content analysis and the method used allows reproducibility of the results. Another strength is that all authors from the interdisciplinary team were involved in the analyses and had unanimous agreement in the categories and coding which limits the risk of bias.

There are some limitations. The study was conducted at a single tertiary care center therefore may not represent patient population of other centers. Similar limitation applies to the demographic distribution. Lastly, the exclusion of non-English speakers and an attrition rate of 17% in responses may result in missing a high-risk subgroups.

Understanding the parents’ experience and the personal, economic, familial, professional, and social (multidimensional) challenges of having a child admitted to the NICU is crucial for identifying coping strategies.

Further studies are needed to develop interventions to alleviate parents’ stress and anxiety and to prove their benefit.

## Data Availability

The original contributions presented in the study are included in the article/Supplementary Material, further inquiries can be directed to the corresponding author.
